# A candidate reference measurement procedure for quantification of glycocholic acid in human serum based on isotope dilution liquid chromatography-tandem mass spectrometry

**DOI:** 10.1007/s00216-024-05449-9

**Published:** 2024-07-24

**Authors:** Pingping Zhang, Huimin Wang, Man Liang, Zhifang Wang, Chunlong Liu, Yanlin Han

**Affiliations:** 1 Reference Laboratory, Autobio Diagnostics Co., Ltd, Zhengzhou, 450016 Henan China; 2 Department of Laboratory Medicine, Affiliated Hospital of Nantong University, Nantong, 226001 Jiangsu China

**Keywords:** Glycocholic acid, Candidate reference measurement procedure, Isotope dilution liquid chromatography-tandem mass spectrometry, Hepatobiliary disease

## Abstract

Accurate measurement of serum glycocholic acid (GCA) is crucial for evaluating the activity of chronic hepatitis. Moreover, GCA is a novel identified biomarker for hepatocellular carcinoma. Although some laboratories have used the liquid chromatography-tandem mass spectrometry (LC–MS/MS) method to measure GCA in recent years, the problem of potential interference of GCA analogues has not been solved well yet. Neither reference measurement procedures nor reference materials for GCA have been listed in the Joint Committee for Traceability in Laboratory Medicine (JCTLM) database. For standardization of GCA, it is urgent to establish a candidate measurement procedure for GCA. In this study, a candidate reference measurement procedure for the quantification of GCA in human serum based on isotope dilution liquid chromatography-tandem mass spectrometry (ID-LC–MS/MS) by a two-step sample pretreatment of protein precipitation and MAX solid-phase extraction was developed and validated. GCA can be completely separated from its structural analogues with gradient elution in 9 min compared with short time gradients published in previous literature by Huang’s group. Method validation indicated perfect quantitation precision with intra-day and inter-day values that were ≤1.30% and ≤1.80%, respectively. The method showed excellent linearity with high regression coefficients (*R*^2^ > 0.999) over a range of 0.92 ng/g–38.38 μg/g and perfect recoveries at three spiked levels (99.87–100.43%). No interference, matrix effect, and carryover were observed. Moreover, the cRMP was successfully applied to measure GCA in serum samples and compared with two immunoassays in a clinical laboratory. As a candidate reference method, this method can promote a GCA standardization program.

**Graphical Abstract:**

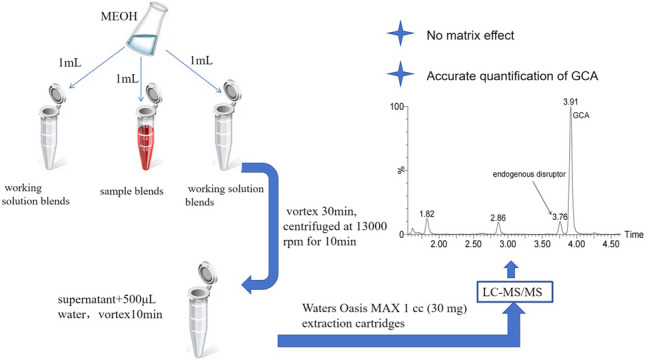

**Supplementary Information:**

The online version contains supplementary material available at 10.1007/s00216-024-05449-9.

## Introduction

Bile acids are steroid acids found predominantly in bile of mammals. Bile acids are physiological detergents that facilitate excretion, absorption, and transport of fats and sterols in the intestine and liver [1–3]. Bile acids that are synthesized from cholesterol in the hepatocyte are termed primary bile acids. Bile acids that are formed by bacterial modification of primary bile acids are termed secondary bile acids [4–6]. Primary bile acids such as cholic acid and chenodeoxycholic acid can be conjugated with glycine or taurine to generate GCA and taurocholic acid [7]. GCA levels were significantly elevated in most patients in all hepatobiliary disease groups [8–11]. In previously published literature, the GCA in urine and blood is an important biomarker for hepatocellular carcinoma (HCC) [12–14]. HCC is the third leading cause of cancer mortality worldwide. Studies have shown that the level of GCA in HCC patients was significantly higher than healthy controls [15]. In addition, GCA was found to be a potential biomarker related to liver cirrhosis [16, 17]. Moreover, several research studies found that the level of serum GCA also provided important information for some diseases [18–21].

Currently, a variety of measurement methods have been developed to detect GCA in serum, which include homogeneous enzyme immunoassay, latex-enhanced immunoturbidimetric method, chemiluminescence immunoassay, high-performance liquid chromatography (HPLC), and gas chromatography-mass spectrometry (GC–MS). Immunoassays are widely applied in clinical laboratory for GCA. However, because of specificity of antibodies, the results of immunoassays may vary greatly in different laboratories. HPLC with different types of detectors (e.g., differential refractometer or ultraviolet detector) has limited specificity and sensitivity disadvantages [22]. GC–MS analysis requires derivatization of analytes usually, which is relatively time-consuming, so it is not suitable for clinical application. The LC/MS/MS method could provide high sensitivity and specific, accurate, and reliable quantitative analysis. A limited number of isotope dilution MS-based methods to measure serum GCA have been reported [22, 23]. However, due to the complexity of serum matrix and a large variety of bile acids in the human serum, a rapid mobile phase gradient cannot completely separate GCA from disruptors. Isotope dilution liquid phase chromatography-tandem mass spectrometry (ID-LC–MS/MS) is recognized as a reference measurement principle; for example, the reference methods for progesterone and testosterone are based on ID-LC/MS/MS. Therefore, there is an urgent need to establish an accurate and specific ID-LC–MS/MS method for GCA.

In the present study, an accurate and sensitive ID-LC–MS/MS method for quantification of GCA was established. GCA can completely separate from endogenous disruptors in the serum with gradient elution in 9 min. The stable isotope-labeled internal standard was used in the pretreatment process to offset the loss of constituents. The bracketing calibration method coupled with the isotope dilution method was used to estimate GCA concentration.

## Materials and methods

### Chemical reagents and serum samples

Chromatographic grade methanol and acetonitrile (ACN) were acquired from Merck (Darmstadt, Germany). Ammonium acetate (LC–MS grade) and chenodeoxycholic acid were purchased from Sigma-Aldrich. De-ionized water prepared by a Milli-Q system (Millipore, MA, USA) was used as a solvent. GCA pure substance and its isotopically labeled internal standard (IS) glycocholic-2,2,4,4-d4 acid (GCA-d4) were obtained from Mikromol (LGC Standards GmbH, Germany) and Toronto Research Chemicals (Toronto, Canada), respectively. Hyodeoxycholic acid, ursodesoxycholic acid, deoxycholic acid, taurocholic acid, cholic acid, and glycochenodexycholic acid were all purchased from MACKLIN (MACKLIN, Shanghai). Glycodeoxycholic acid was obtained from Aladdin (Aladdin, Shanghai). Glycoursodeoxycholic acid was obtained from RHAWN (RHAWN, Shanghai). The mixed-mode anion exchange Oasis MAX SPE cartridges (1 cc, 30 mg) were purchased from Waters. The weights of the samples and standard solutions were performed on a Mettler Toledo XPE205 balance with the readability of 0.01 mg. The serum for the method validation and comparison was obtained from the remaining samples of the company’s kit study.

### Preparation of calibrators and internal standard

Standard stock solutions of GCA were prepared by weighing approximately 20 mg of GCA pure substance then the GCA pure substance was dissolved in methanol. The mass of methanol used was also accurately measured. The concentration of the stock solution was approximately 284 μg/g. The working solutions were prepared to be approximately 19.21 μg/g (WS-A), 1.757 µg/g (WS-B), and 174 ng/g (WS-C) by dilution of stock solution. Stock solutions of GCA-D4 were prepared by dissolving 10 mg (purity, 98%) GCA-D4 pure substance in 5 mL 50% methanol/water (v/v) to obtain concentration of 1.96 mg/mL. The internal standard working solution (IS-WS) was prepared by gradually diluting stock solutions of GCA-D4 with 50% methanol. IS-WS-A solutions (19.6 μg/mL), IS-WS-B solutions (1.96 μg/mL), and IS-WS-C solutions (0.196 μg/mL) were prepared according to the above method in a 100-mL volumetric flask, respectively. Due to the relatively wide detection range, WS-A/ISWS-A, WS-B/ISWS-B, and WS-C/ISWS-C solutions were used to quantitate samples with GCA concentration ≤ 200 ng/mL, 200–2000 ng/mL, and ≥ 2000 ng/mL, respectively.

### Sample pretreatment

Serum samples were allowed to equilibrate to room temperature. For analysis, 50 μL IS-WS was gravimetrically added to the serum to get an approximately 1:1 mass ratio of analyte to IS. Then 1 mL methanol was slowly added to the sample blends to precipitate protein, while mixing gently on a vortex mixer for 30 min. The sample blends were then centrifuged at 13,000 rpm for 10 min. The resulting supernatant was transferred into a 5-mL centrifuge tube which was added 500 μL water initially, then vortexed for 10 min. The above mixture was transferred to Waters Oasis MAX 1 cc (30 mg) extraction cartridges which were preconditioned with methanol (1 mL) followed by water (1 mL). The loaded cartridges were washed sequentially with water containing 5% ammonium hydroxide (1 mL) and methanol (1 mL). The GCA was eluted from the cartridge with 1 mL methanol containing 2% formic acid. The eluate was dried under nitrogen at room temperature and reconstituted with 200 μL 25% acetonitrile/water (v/v).

Fifty microliters of IS-WS was added into gravimetrically standard working solution to obtain mass ratios of GCA to the IS of 0.8 and 1.2, respectively. The working solution blends were pretreated as the above sample pretreatment.

### LC–MS/MS analysis

Chromatographic separation was achieved with the use of a C18 column (ACQUITY UPLC® BEH, 1.7 μm, 100 mm × 2.1 mm) at 40 °C with 1 mM ammonium acetate in water (mobile phase A) and acetonitrile (mobile phase B) at a rate of 0.3 mL/min. Gradient elution condition was as follows: 0–1.0 min, 25% mobile phase B; 1.0–4.0 min, from 25 to 32.5% mobile phase B; 4.0–6.09 min, from 32.5 to 90% mobile phase B; 6.09–7.0 min, 90% mobile phase B; 7.10–9.0 min, 25% mobile phase B. The injection volume was 5 μL and the temperature of the autosampler was set at 10 °C.

A Waters ACQUITY UPLC® system with a triple-quadruple mass detector (Xevo TQ-S) in negative electrospray ionization mode was used for analysis. The transitions and conditions were as follows: m/z 464.39 → 74.28 (quantification) and m/z 464.39 → 402.53 (confirmation) for GCA, and m/z 468.42 → 74.28(quantification) and m/z 468.42 → 406.49 (confirmation) for the internal standard. The optimized instrumental settings were a capillary voltage of 1.9 kV, a desolvation temperature of 600 °C, a cone gas flow rate of 150 L/h, and a desolvation gas flow rate of 1000 L/h.

### Method validation

Performances of the established ID-LC–MS/MS method including intra-assay and inter-assay imprecision, accuracy, linearity, potential interference, matrix effect, and carryover were validated.

### Uncertainty evaluation

The uncertainty was evaluated according to the *ISO Guide to the Expression of Uncertainty in Measurement*. We evaluated the potential sources of uncertainty to calculate the standard uncertainty and expanded uncertainty. For type A evaluation, the standard uncertainty is calculated from repeated independent measurements. For type B evaluation, the standard uncertainty is calculated based on the impurity of reference material, the calibrated weighing data, and the measurement of the serum density. For calculation of the expanded uncertainty, the standard uncertainty was multiplied by the coverage factor *k* = 2, at the 95% confidence level. Low, medium, and high levels of serum samples were used for the assessment. Samples were measured in five replicates on each day for 3 days.

### Method comparison

Serum samples (*n* = 49) with GCA concentrations between 0.023 and 85.165 μg/mL were analyzed by two immunoassays and the ID-LC–MS/MS method. The two immunoassays were the latex-enhanced immunoturbidimetric method and the chemiluminescence immunoassay method.

## Results and discussion

### Optimization of chromatographic conditions

**Fig. 1:**
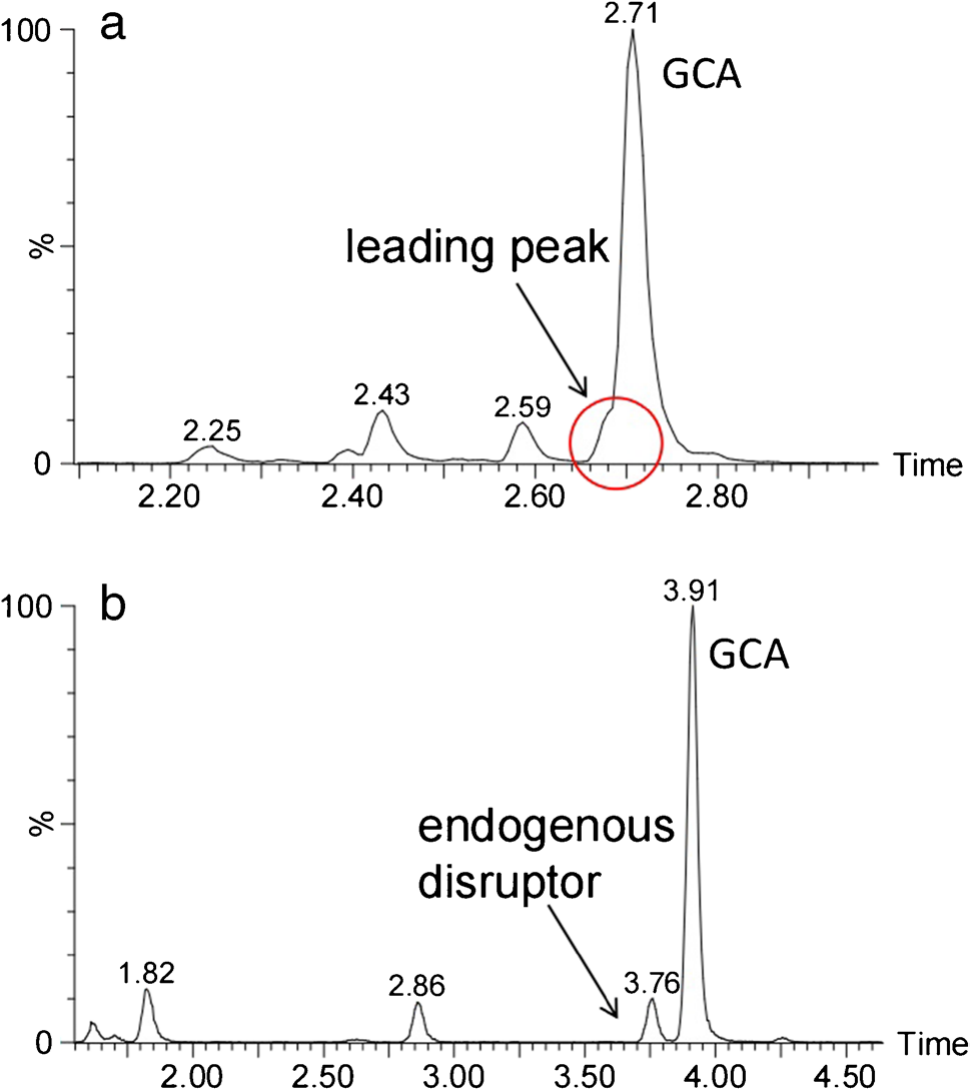
The chromatography of GCA in human plasma in the liquid phase condition A (**a**) and condition B (**b**)

In this study, the liquid phase condition (condition A) of clinical rapid LC–MS/MS method [22] was compared with the liquid phase condition (condition B) of our established candidate reference method (see Supplemental Tables 1–2). Thirty-eight human serum samples were taken for the chromatographic condition study. Then GCA was extracted by the above pretreatment method which was mentioned in the sample preparation part. As can be seen from Fig. 1a, there was a leading peak near GCA peak. But from Fig. 1b, we isolated an unknown endogenous disruptor. The GCA measurement results which were obtained under condition A were generally 3.1 to 20.3% higher than those measured under condition B. We quantified the GCA peak and the endogenous disruptor in condition B, respectively, and found that the bias between the sum of quantitative results of two peak areas under condition B with the quantitative results under condition A was − 2.6 to 3.5% (see supplementary files). There was a significant reduction in bias. It may be implied that the GCA peak under the rapid liquid phase condition was not completely separated from the disruptor in the serum sample, influencing accurate quantification. In addition, the content of endogenous disruptor in serum of patients with hepatobiliary disease was higher than that in the normal group in our study (see supplementary files). This endogenous disruptor was speculated to positively correlate with the concentration of glycocholic acid.

### Selection of protein precipitator

The majority of the GCA in human serum is bound to proteins, so a protein-precipitation approach is required to release GCA from proteins. We studied three different precipitants, including methanol, acetonitrile, and ethanol. Sextuplicate 100-µL aliquots of the human serum from three individuals and GCA working solution (174 ng/g) were added to 2-mL centrifuge tubes containing an appropriate amount of GCA-d4, respectively. One milliliter of protein precipitation was added. The samples were processed as described in the above sample preparation section.

**Fig. 2:**
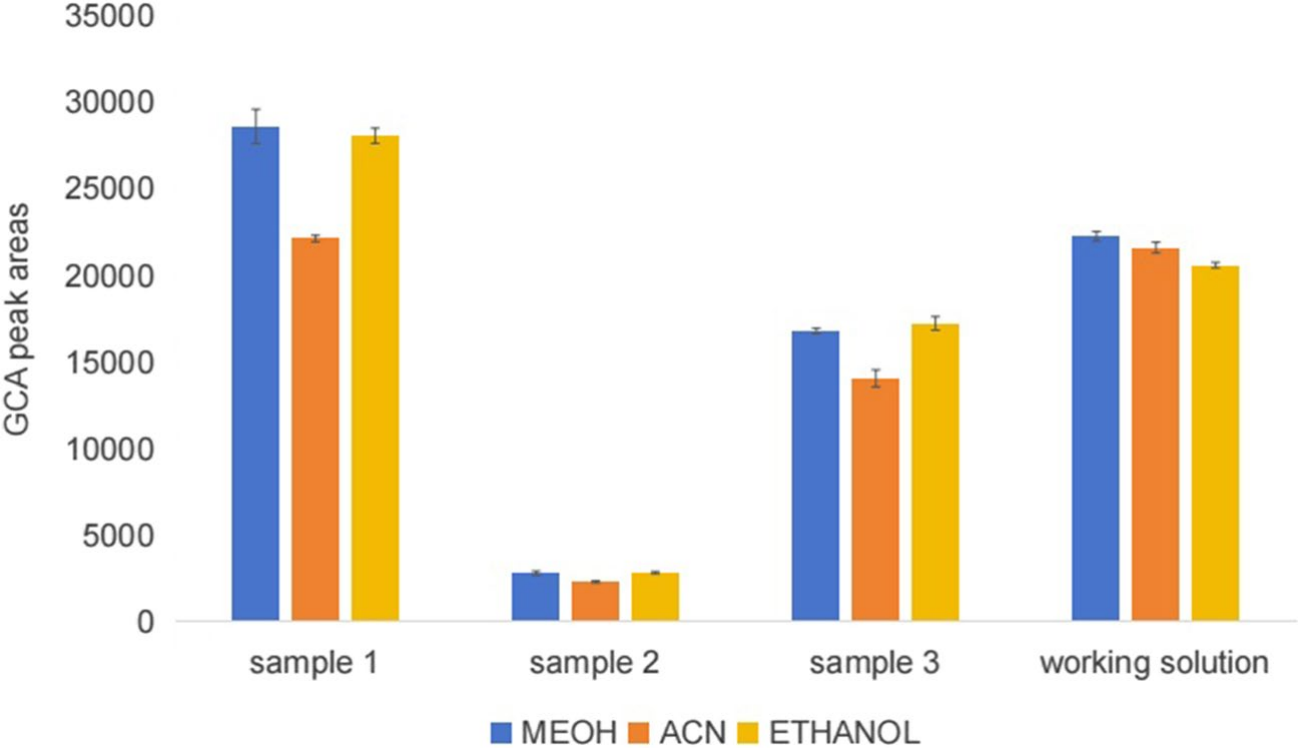
GCA peak areas of different protein precipitators

Figure 2 shows bar graphs of the measured peak areas of GCA detected in human serum blends and standard working solution blends by three protein precipitators. Through the above three protein precipitators, bar graphs of the measured peak areas of GCA are almost the same. In this study, we select methanol as the protein precipitator.

### Sample pretreatment study

Three individual human sera and GCA’s working solution (174 ng/g) were selected to explore the approaches of pretreatment. Serum samples and working solution were allowed to equilibrate to room temperature. In this study, we evaluated three different sample preparation approaches for the extraction of GCA in human serum. For all of the methods 1–3 described below, the final residue after evaporation to dryness was dissolved in 200 μL of the HPLC mobile phase and kept at 10 ℃. A 5-μL aliquot was injected for LC/MS/MS analysis.

#### Method 1: Protein precipitation only

One hundred-microliter aliquots of the human serum test sample and GCA’s working solution (174 ng/g) in triplicate were added to 2-mL centrifuge tubes containing the same amount of GCA-d4 for sample preparation, respectively. Then 1 mL methanol was slowly added to the 2-mL centrifuge tube, while mixing gently on a vortex mixer at 2000 rpm for 20 min. The sample solution was then centrifuged at 13,000 rpm for 10 min. The 500-μL supernatant liquid was transferred into a 15-mL centrifuge tube and evaporated to dryness under a constant stream of nitrogen.

#### Method 2: Deproteinization and use of solid-phase extraction (SPE) Oasis HLB cartridges

The portion of protein precipitation in front is the same as that in method 1. The 500-μL supernatant liquid was transferred into a 5-mL centrifuge tube which was added 1500 μL water initially. Next, the supernatant mixture is mixed in a vortex mixer. The SPE cartridge was conditioned by washing with 1 mL of methanol followed by 1 mL of water. The serum supernatant mixture and working solution supernatant mixture were then immediately applied to Waters Oasis HLB 1 cc (30 mg) extraction cartridges. The cartridges were then washed with 1 mL of 5:95 methanol/water. Finally, the cartridges were eluted with 500 μL of methanol which were collected and evaporated to dryness under a stream of nitrogen.

#### Method 3: Deproteinization and use of solid-phase extraction (SPE) Oasis MAX cartridges

The portion of protein precipitation in front is the same as that in method 1. The 500-μL supernatant liquid was transferred into a 5-mL centrifuge tube which was added 1500 μL water initially. Next, the supernatant mixture is mixed in a vortex mixer. The SPE cartridge was conditioned by washing with 1 mL of methanol followed by 1 mL of water. The serum supernatant mixture and working solution supernatant mixture were then immediately applied to Waters Oasis MAX 1 cc (30 mg) extraction cartridges. The cartridges were then washed with 1 mL of a 5% aqueous solution of NH_4_OH followed by 1 mL methanol. Finally, the cartridges were eluted with 500 μL of methanol containing 2% formic acid which were collected and evaporated to dryness under a stream of nitrogen.

**Fig. 3:**
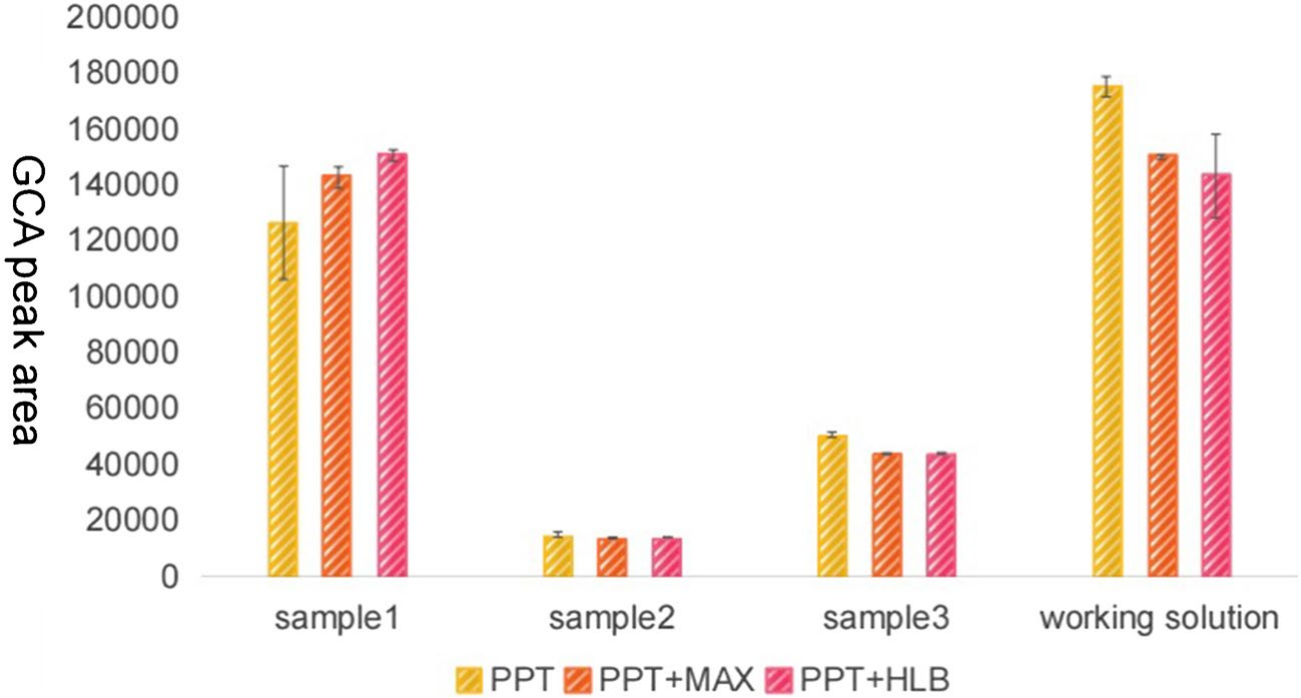
GCA peak areas of different pretreatment methods

**Fig. 4:**
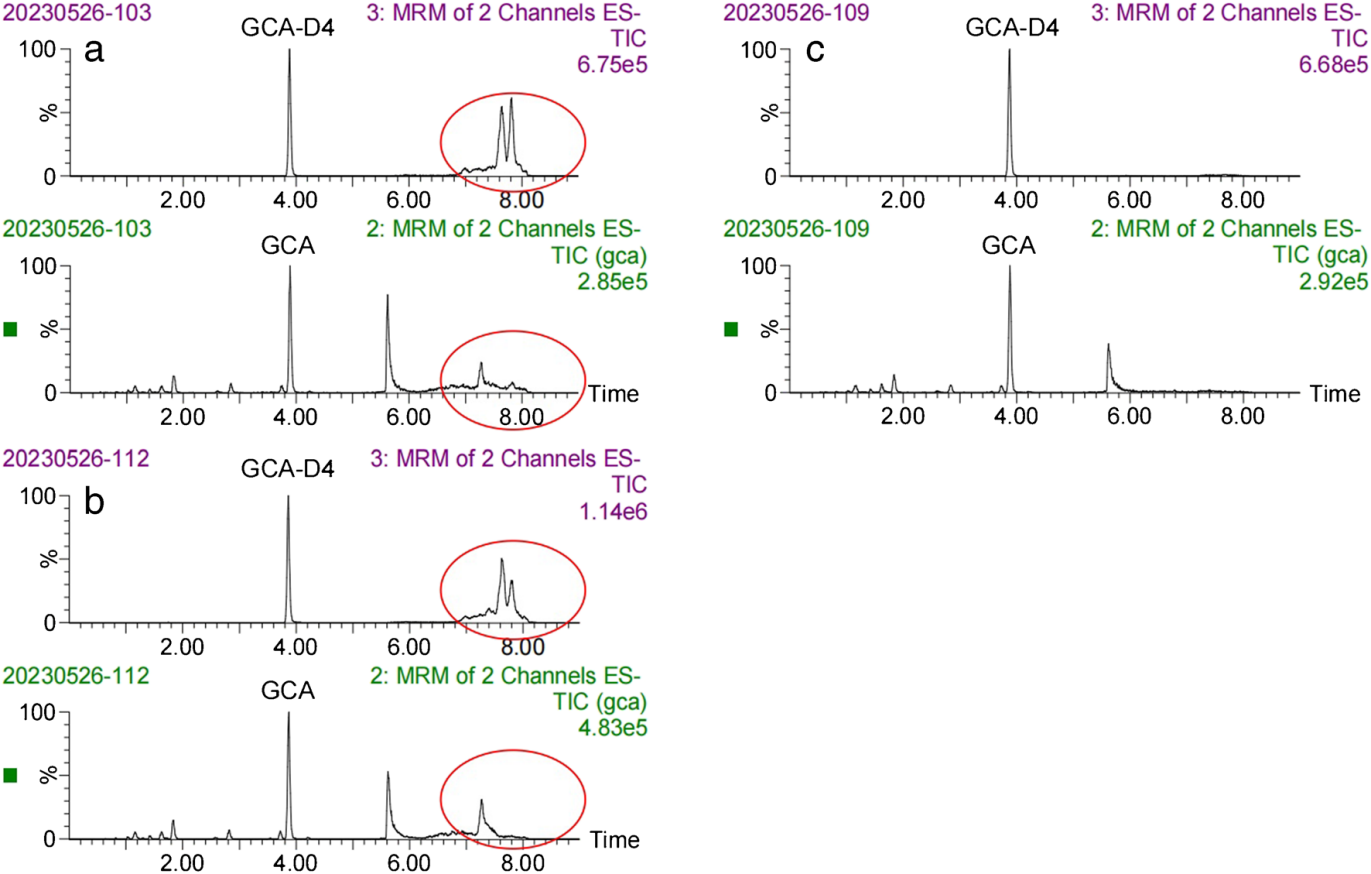
The chromatogram for three pretreatment methods: **a** method 1, **b** method 2, **c** method 3

We studied three pretreatment methods, including protein precipitation only (method 1), deproteinization and use of solid-phase extraction (SPE) Oasis HLB cartridges (method 2), and deproteinization and use of solid-phase extraction (SPE) Oasis MAX cartridges (method 3). Figure 3 shows bar graphs of the measured peak areas of GCA detected in human serum blends and standard working solution blends by three approaches of pretreatment. Through the above three pretreatment methods, bar graphs of the measured peak areas of GCA are almost the same (*p* value > 0.05, *T* test). But from the chromatogram (Fig. 4), when using method 3, the chromatogram of method 3 was purer than method 1 and method 2. Finally, we chose method 3 as the optimal sample pretreatment method.

### Optimal equilibration method

The equilibration method is a critical parameter for accurate measurement. Eight 100-µL aliquots from two individual sera were taken for the equilibration study, respectively. Human serum materials were added to tubes containing an appropriate amount of GCA-d4. The mixtures which were equilibrated at room temperature were divided into four groups as shown in Table 1. Then the samples were processed as described in the sample preparation section.

**Table 1:** Equilibration methods exploration

Groups	Equilibration methods
1	After vortex oscillating for 20 min, add precipitant
2	After vortex oscillating for 30 min, add precipitant
3	After vortex oscillating for 60 min, add precipitant
4	Add precipitant first and then vortex for 30 min

The result (Table 2) showed that the ratio of four groups was basically the same, and the ratio of GCA to GCA-d4 remained unchanged until 60 min. Therefore, we finally chose the fourth group for sample equilibration.

**Table 2:** The ratio of GCA to GCA-d4 in different groups

Sample	Group 1	Group 2	Group 3	Group 4
Sample 1	1.032	1.041	1.042	1.045
Sample 2	1.000	0.999	1.003	1.008

### Linearity analysis

**Fig. 5:**
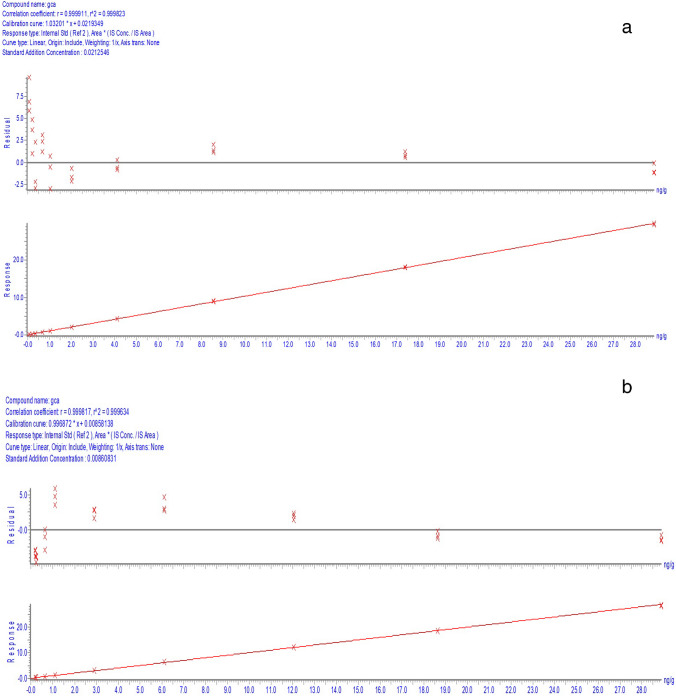
The graphs of GCA’s linear analysis (**a**, 0.92–395.02 ng/g; **b**, 0.25–38.38 μg/g)

The method linearity was assessed by adding different amounts of GCA standard with the sample amount of IS-WS to blank matrix (3% bovine serum albumin solution). Each sample was measured three times. The ratios of GCA to GCA-D4 area counts were plotted against mass ratios. Linear responses were observed between 0.92 and 395.02 ng/g from the plot derived from the equation *y* = 1.032*x* + 0.0219 (*R*^2^ = 0.9998) and between 0.25 and 38.38 μg/g from the plot derived from the equation *y* = 0.996872*x* + 0.00858 (*R*^2^ = 0.9996). The graphs of GCA’s linear analysis are shown in Fig. 5.

### The limit of detection (LOD) and the limit of quantification (LOQ)

The limit of detection (LOD) was defined as the GCA concentration at which the signal to noise (S/N) ratio was ≥ 3, and the limit of quantification (LOQ) was defined as the GCA concentration at which the S/N ratio was ≥ 10, CV ≤ 20.0%, and the biases against target values ≤ 15.0%. The LOD and LOQ were determined by the measurements of samples prepared with 3% bovine serum albumin solution spiked with GCA at various concentrations to be 0.082 ng/g (S/N = 14) and 0.676 ng/g (S/N = 137, CV = 4.34%, bias = 5.33%, *n* = 6).

### Matrix effect

The matrix effect was estimated by measuring seven different native serum samples mixed with GCA standard solutions in several proportions, 0%, 20%, 50%, 80%, and 100%, respectively. The same amount of internal standard solutions were added and the peak area ratios (PARs) of GCA/IS were measured. The percent matrix biases against the expected value based on the proportion of each sample in a mixture were calculated for the evaluation of matrix effect. The equations were as follows:

- 20% matrix bias = [C − (0.8A + 0.2B)] / (0.8A + 0.2B) × 100%
- 50% matrix bias = [D − (0.5A + 0.5B)] / (0.5A + 0.5B) × 100%
- 80% matrix bias = [E − (0.2A + 0.8B)] / (0.2A + 0.8B) × 100%

The percent matrix biases were 0.17%, 0.72%, and 0.06% at the proportions of 20%, 50%, and 80%, shown in Table 3.

**Table 3:** The result of percent matrix bias

	PAR of 0% matrix (A)	PAR of 100% matrix (B)	PAR of 20% matrix (C)	PAR of 50% matrix (D)	PAR of 80% matrix (E)
Sample 1	1.015	0.941	0.981	0.968	0.952
Sample 2	0.998	1.646	1.140	1.331	1.531
Sample 3	0.988	1.087	0.993	1.066	1.071
Sample 4	0.994	0.657	0.932	0.830	0.732
Sample 5	1.009	2.603	1.337	1.798	2.245
Sample 6	1.007	1.644	1.136	1.314	1.519
Sample 7	1.005	7.46	2.318	4.305	6.194
Average percent matrix bias	/	/	0.17%	0.72%	0.06%

### Interference

To assess interference, we performed analysis on low, medium, and high human serum samples which were spiked with bilirubin, hemoglobin, or lipids, respectively. The recoveries of GCA ranged from 99.01 to 101.30% for samples containing bilirubin up to 0.2 mg/mL, 99.68 to 100.00% for hemolyzed samples with hemoglobin up to 5 mg/mL, and 98.70 to 100.11% in the presence of triglycerides up to 30 mg/mL, respectively, shown in Table 4.

**Table 4:** The recoveries of GCA for samples containing the potential interferences

Sample	Expected value (μg/g)	Bilirubin		Hemoglobin		Triglycerides	
		Detected value (μg/g)	Recovery (%)	Detected value (μg/g)	Recovery (%)	Detected value (μg/g)	Recovery (%)
Sample 1	0.077	0.078	101.30%	0.077	100.00%	0.076	98.70%
Sample 2	1.753	1.770	100.97%	1.751	99.89%	1.755	100.11%
Sample 3	12.366	12.243	99.01%	12.326	99.68%	12.344	99.82%

### Carryover study

The level of carryover by the autosampler sampling process was evaluated by measuring samples with GCA at 46 μg/g and 0.4 ng/g. We performed measurements with the samples in sequences of 0.4 ng/g, 0.4 ng/g, 46 μg/g, and 0.4 ng/g. The carryover rate was < 0.01%.

### Trueness and analytical recovery

At present, there are no RMP or certified RMs (CRMs) developed for GCA in JCTLM database; herein, trueness of the LC–MS/MS method was assessed by spiking recovery experiment. The samples spiked with GCA standard solution at three different concentrations (200 ng/g, 488 ng/g, 2973 ng/g) were used to recovery analysis. The samples were analyzed in triplicate in two runs. The recovery of each sample ranged from 99.87 to 100.43%, shown in Table 5.

**Table 5:** The recovery of added GCA

Added value (ng/g)	Detected value (ng/g)	Expected value (ng/g)	Recovery (%)	CV (*n* = 6, %)
0	205	/	/	0.60%
200	405	405	100%	0.73%
488	696	693	100.43%	0.99%
2973	3174	3178	99.87%	1.34%

### Precision

The precision of measurements was evaluated, by analyzing five replicates of three samples at three concentrations over three runs. The results are shown in Table 6, for within-run imprecision ranging from 0.70 to 1.30% and intermediate measurement imprecision ranging from 0.70 to 1.80%.

**Table 6:** Imprecision of measurements of serum GCA by LC/MS/MS

Sample	Run	Mean (μg/g)	Overall mean (μg/g)	Within-run CVs (%)	Intermediate measurement CVs (%)
Sample 1	1	0.080	0.079	1.30	1.80
	2	0.078			
	3	0.079			
Sample 2	1	1.816	1.804	1.01	1.10
	2	1.802			
	3	1.794			
Sample 3	1	12.287	12.303	0.70	0.70
	2	12.294			
	3	12.328			

### Specificity

**Fig. 6:**
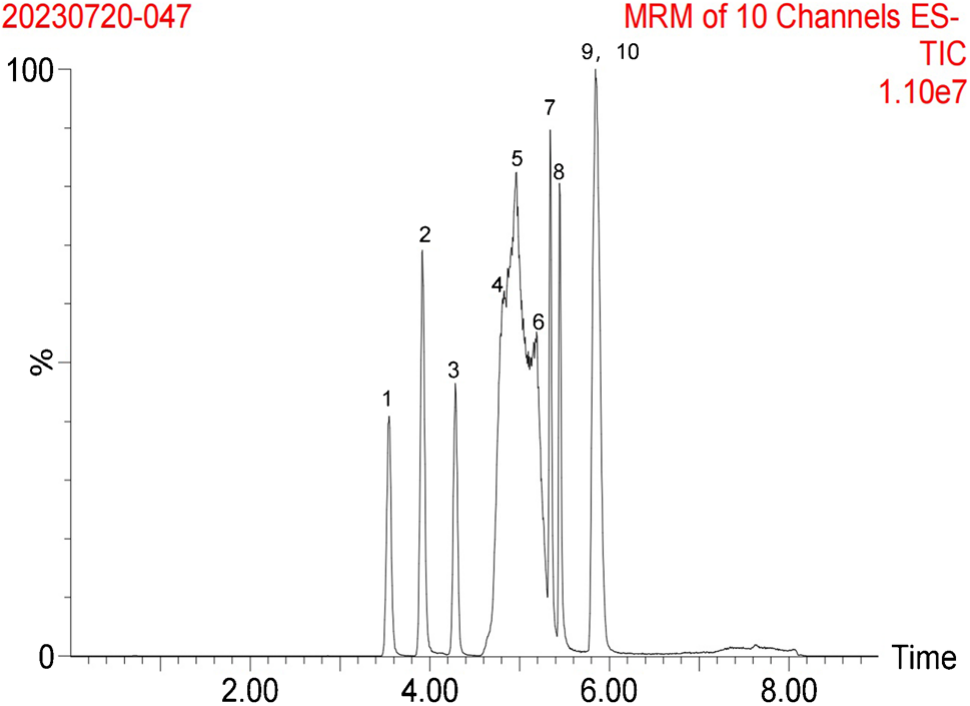
The total ion chromatogram (TIC) for all the structural analogues

To investigate analytical specificity, the metabolites and structural analogues of GCA, such as cholalic acid (CA), taurocholic acid (TCA), deoxycholic acid (DCA), chenodeoxycholic acid (CDCA), hyodeoxycholic acid (HDCA), ursodeoxycholic acid (UDCA), glycodeoxycholic acid (GDCA), glycochenodeoxycholic acid (GCDCA), and glycoursodeoxycholic acid (GUDCA), were analyzed simultaneously. Nine structural analogues of GCA listed in Table 7 did not contain the same mass transitions of GCA. Moreover, no identifiable peaks with the same retention time as that of GCA was observed. The liquid chromatography conditions of this method allow complete baseline resolution of GCA from interferences (Fig. 6).

**Table 7:** Analogues used for selectivity analysis

Number	Analogues	Molecular mass (g/mol)	Ionization mode	Q1 mass (Da)	Q3 mass (Da)
2	GCA	465.62	ESI-	464.39	74.28
4	CA	408.57	ESI-	407.41	289.25
3	TCA	515.7	ESI-	514.41	80.04
9	DCA	392.58	ESI-	391.35	345.29
10	CDCA	392.57	ESI-	391.35	391.37
6	HDCA	392.57	ESI-	391.3	391.3
5	UDCA	392.57	ESI-	391.3	391.3
8	GDCA	467.64	ESI-	448.3	74.09
7	GCDCA	449.62	ESI-	448.3	74.16
1	GUDCA	449.62	ESI-	448.3	74.16

### Measurement uncertainty

The relative expanded uncertainties at concentrations of 0.1735, 3.933, and 26.97 μmol/L were 2.0%, 1.3%, and 0.9%, respectively.

### Method comparison

**Fig. 7:**
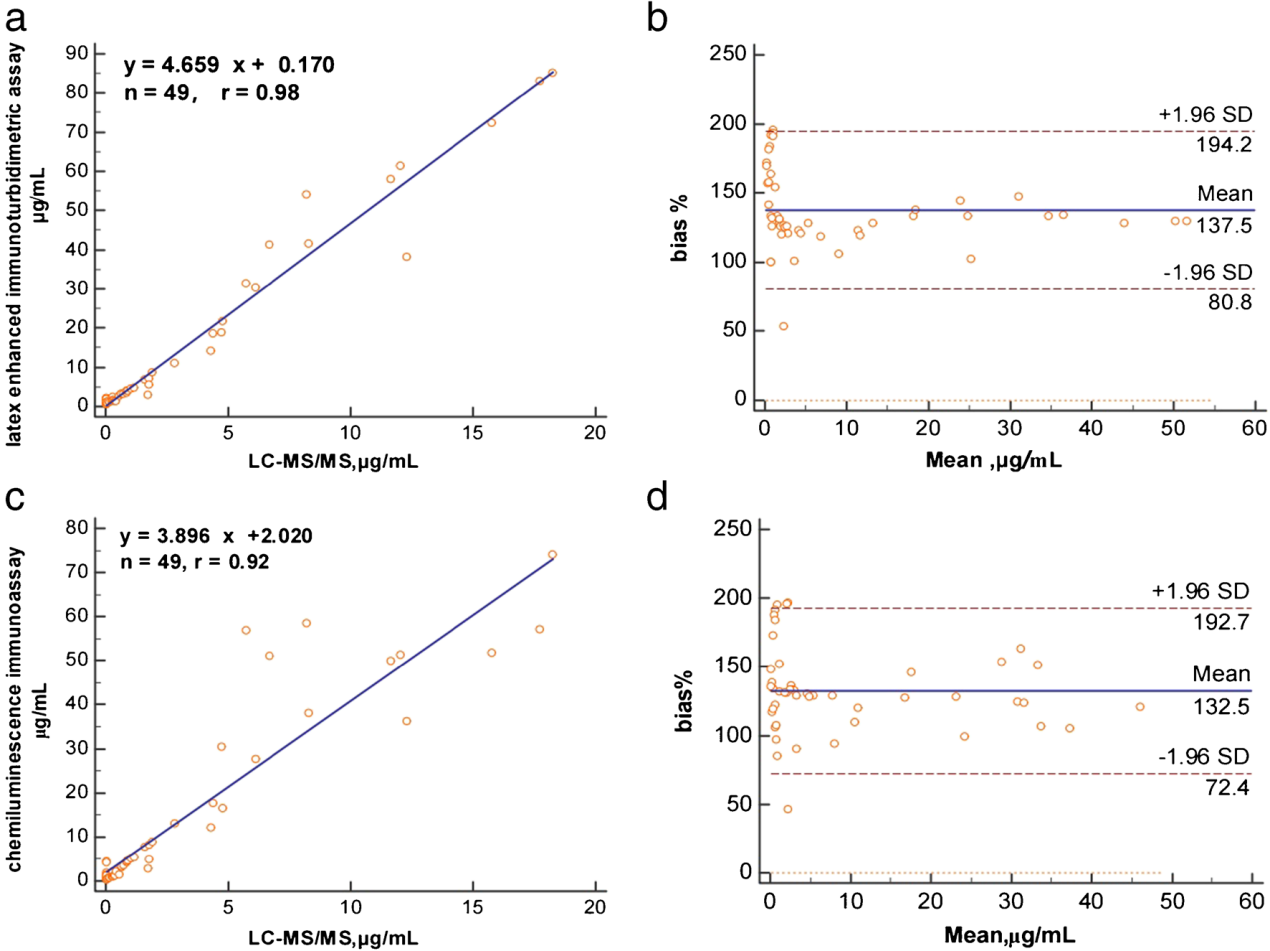
Correlation (**a**) and Bland–Altman plots (**b**) of GCA concentrations between the LC–MS/MS method and latex-enhanced immunoturbidimetric method. Correlation (**c**) and Bland–Altman plots (**d**) of GCA concentrations between the LC–MS/MS method and chemiluminescence immunoassay method

The overall results of the cRMP and two immunoassays are compared in Fig. 7. Linear regression and Bland–Altman analysis were used to evaluate the results. Figure 7 shows linear regression *r* = 0.98 and *r* = 0.92, respectively. The Bland–Altman plot demonstrated significant positive bias. This results from cross-reactivities of immunoassays with other bile acids in serum [24].

## Conclusion

In this study, different pretreatment methods were compared, and then we identified a pretreatment without significant matrix effects based on protein precipitation and MAX solid-phase extraction. The liquid phase gradient which was compared with rapid gradients published in previous literature can completely separate glycolic acid from an unknown endogenous interferor. Our data show that this unknown endogenous disruptor is significantly correlated with the bias of results under the two liquid phase conditions. At the same time, this unknown endogenous disruptor is also significantly different in normal group and patients with liver disease. This endogenous disruptor was speculated to positively correlate with the concentration of glycocholic acid. Further research is needed. The method were sufficiently validated. No interference, matrix effect, and carryover were observed. The comparison results between different methods indicate that the current immunoassays vary greatly. The established cRMP can be used for measurement traceability, and it provides an accuracy base to routine methods for GCA.

## Supplementary Information

Below is the link to the electronic supplementary material.

ESM 1 (PDF 120 KB)

ESM 2 (XLS 21.5 KB)

ESM 3 (XLS 53.5 KB)

ESM 4 (XLS 27.0 KB)

## Abbreviations

GCA: Glycocholic acid
GCA-d4: Glycocholic-2,2,4,4-d4 acid
HCC: Hepatocellular carcinoma
cRMP: Candidate reference measurement procedure
CV: Coefficient of variation
JCTLM: Joint Committee for Traceability in Laboratory Medicine
LC–MS/MS: Liquid chromatography-tandem mass spectrometry
ID-LC–MS/MS: Isotope dilution liquid chromatography-tandem mass spectrometry
LOD: Limit of detection
LOQ: Limit of quantification
MRM: Multiple reactive monitoring
HPLC: High-performance liquid chromatography
GC-MS: Gas chromatography-mass spectrometry
UPLC: Ultra-performance liquid chromatography
