# Supplementary material for: A candidate reference measurement procedure for quantification of glycocholic acid in human serum based on isotope dilution liquid chromatography-tandem mass spectrometry
Source: Anal Bioanal Chem. 2024 Jul 24;416(23):5121–31. doi: 10.1007/s00216-024-05449-9 (PMC11377629; doi:10.1007/s00216-024-05449-9)
Supplement: Supplementary file 1 — (PDF 120 KB) [file 216_2024_5449_MOESM1_ESM.pdf]

A candidate reference measurement procedure for quantification of glycocholic acid in human serum based on isotope dilution liquid chromatography-tandem mass spectrometry

Pingping Zhang[1] · Huimin Wang[2] · Man Liang [1] · Zhifang Wang [1] ·Chunlong Liu[1] · Yanlin Han [1]

1 *Reference Laboratory, Autobio Diagnostics Co., Ltd, Zhengzhou, Henan, 450016, China*

2 *Department of Laboratory Medicine, Affiliated Hospital of Nantong University, Nantong, Jiangsu, 226001, China*

Corresponding author: Yanlin Han

Email: hanyanlin@autobio.com.cn

Journal name：Analytical and Bioanalytical Chemistry

Supplemental Table 1. liquid phase condition A gradient at a flow rate of 0.300 mL/min

Time (min)	% A (0.2 mM ammonium acetate containing 0.05% formic acid in water)	% B (acetonitrile containing 0.05% formic acid)
0	70	30
1	70	30
2.5	10	90
3.5	10	90
3.6	70	30
4.0	70	30

Supplemental Table 2. liquid phase condition B gradient at a flow rate of 0.300 mL/min

Time (min)	% A (1 mM ammonium acetate in water)	% B (100% acetonitrile)
0	75	25
1	75	25
4	67.5	32.5
6.09	10	90
7	10	90
7.1	75	25
9	75	25
